# Multifactorial Causes and Consequences of TLSP Production, Function, and Release in the Asthmatic Airway

**DOI:** 10.3390/biom14040401

**Published:** 2024-03-26

**Authors:** Danica L. Brister, Hafsa Omer, Christiane E. Whetstone, Maral Ranjbar, Gail M. Gauvreau

**Affiliations:** Division of Respirology, Department of Medicine, McMaster University, Hamilton, ON L8N 3Z5, Canada; bristerd@mcmaster.ca (D.L.B.); omerh@mcmaster.ca (H.O.); whetstoc@mcmaster.ca (C.E.W.); ranjbm1@mcmaster.ca (M.R.)

**Keywords:** asthma, thymic stromal lymphopoietin, airway epithelium

## Abstract

Disruption of the airway epithelium triggers a defensive immune response that begins with the production and release of alarmin cytokines. These epithelial-derived alarmin cytokines, including thymic stromal lymphopoietin (TSLP), are produced in response to aeroallergens, viruses, and toxic inhalants. An alarmin response disproportionate to the inhaled trigger can exacerbate airway diseases such as asthma. Allergens inhaled into previously sensitized airways are known to drive a T2 inflammatory response through the polarization of T cells by dendritic cells mediated by TSLP. Harmful compounds found within air pollution, microbes, and viruses are also triggers causing airway epithelial cell release of TSLP in asthmatic airways. The release of TSLP leads to the development of inflammation which, when unchecked, can result in asthma exacerbations. Genetic and inheritable factors can contribute to the variable expression of TSLP and the risk and severity of asthma. This paper will review the various triggers and consequences of TSLP release in asthmatic airways.

## 1. Introduction

The airway epithelium forms a continuous and regulated barrier that covers the airway lumen and forms the first line of defense against pathogens, allergens, noxious fumes, and inhaled particulates. The response of the epithelium to these diverse insults forms the basis of both health and disease. When the airway epithelium interacts with these triggers, alarmin cytokines including interleukin (IL)-25, IL-33, and thymic stromal lymphopoietin (TSLP) are produced by epithelial cells and bind to receptors found on intraepithelial immune cells as well as those located in the submucosa, circulatory bloodstream, and distant sites. By signaling through the TSLP heterodimer receptor comprised of TSLPR (TSLP receptor) and IL-7Rα (IL-7 receptor alpha), TSLP induces the development and expansion of cells of both the innate and adaptative immune system [1]. In the asthmatic airway, the alarmin response is exaggerated [2,3]. In this review, we summarize the evidence of excessive TSLP production in airway epithelial cells in those with asthma, as well as how various important triggers have been shown stimulate TSLP production, leading to clinical consequences for asthmatic patients.

## 2. Airway Epithelium

### 2.1. Airway Epithelium Forms the First Line of Defense

The structural integrity of the airway epithelium is paramount for tissue defense. The bronchial epithelium is comprised of a pseudostratified layer of basal cells, columnar ciliated cells, goblet cells, and club cells [4], which form a defensive barrier and secrete mucous, limiting access to the epithelium. In asthmatic patients, there is evidence of epithelial cell hypertrophy, mucous metaplasia, and ciliary dysfunction, resulting in abnormal mucous production and clearance [5]. Over time, asthma remodeling, characterized by smooth muscle cell hyperplasia and hypertrophy, together with airway wall thickening, can lead to fixed obstruction and intractable symptoms [5].

**Figure 1:**
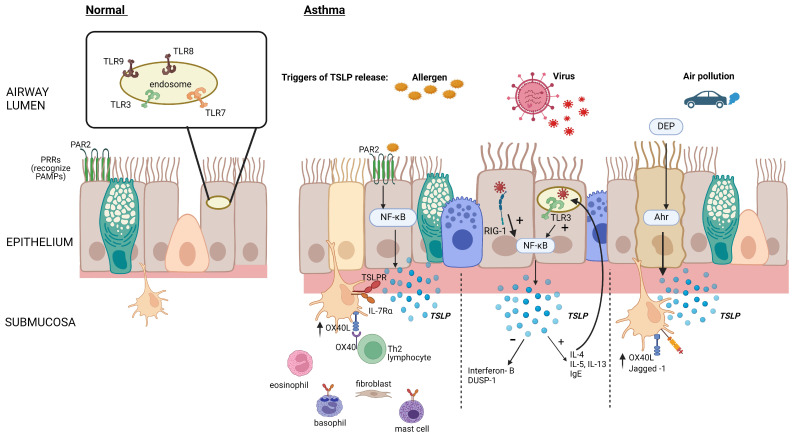
Epithelial TSLP production in response to inhaled triggers. Allergen activation of epithelial PRRs such as protease-activated receptor (PAR2) stimulates the production of TSLP via nuclear factor kappa B (NFκB) activation, leading to increased OX40 ligand (OX40L) expression on dendritic cells and initiation of the type 2 (T2) inflammatory cascade. Viral components are recognized by cytosolic RNA helicases such as retinoic acid–inducible gene (RIG-1), as well as by toll-like receptor (TLR)-3 in epithelial cell endosomes, increasing the production of TSLP and reducing the production of interferons and dual-specificity protein phosphatase 1 (DUSP-1). Diesel exhaust particles (DEP) augment the binding of the aryl hydrocarbon (Ah)/aryl hydrocarbon receptor (Ahr) nuclear translocator complex to gene promoter regions, thereby increasing the production of TSLP. Created with BioRender.com.

The detection of pathogens is a fundamental function of the epithelial barrier. Proteins or lipids from these organisms, viral DNA, and allergens are detected by transmembrane or intercellular pattern recognition receptors (PRRs) that recognize highly conserved microbial motifs called pathogen-associated molecular patterns (PAMPs) and host-derived molecular damage-associated molecular patterns (DAMPs) [6] (Figure 1). These cell-bound receptors are divided into secreted receptors, cytosolic DNA receptors, and intracellular cytosolic receptors and can initiate host defense through the rapid production of the alarmin cytokines TSLP, IL-25, and IL-33 [7,8]. These receptors can also be triggered by other pro-inflammatory cytokines and proteases [7,8].

### 2.2. Elevated TSLP Expression in Asthmatic Airways

There are differences in the level of TSLP expression by epithelial cells in individuals with asthma, atopy, and other allergic diseases compared to healthy controls [2]. Under baseline conditions, endobronchial tissue from asthmatic patients has a higher number of airway epithelial cells expressing TSLP mRNA compared to normal controls [2]. In addition, the level of TSLP in the airways of asthmatic individuals is positively correlated with disease severity [9]. In response to viral or fungal stimuli, airway epithelial cells from individuals with asthma release even greater amounts of TSLP, [9,10,11,12] thereby polarizing T helper 2 (Th2) cells and expanding innate lymphoid (ILC2) cells to enhance the production of T2 cytokines, including IL-4, IL-13, and IL-5 [13,14,15]. 

More recent evidence has identified two TSLP isoforms in human tissues; the short-form TSLP (sf TSLP) is found in healthy cells under homeostatic conditions and the long-form TSLP (lf TSLP) is the main transcript variant produced in response to inflammatory and mechanical triggers [16]. Current assays for TSLP protein levels do not differentiate between these isoforms because they share identical c-terminus amino acid sequences, but their distinct promoter regions allow them to be identified by mRNA quantification [17]. sfTSLP does not activate the TSLPR receptor, and its function remains unclear; however, a recent study using a mouse model suggested that sfTSLP might mitigate inflammation in asthma by impeding the binding of lfTSLP to its receptor complex [18]. In human skin, sfTSLP has demonstrated antimicrobial properties, providing further evidence of its immunoregulatory potentials [19]. Given that the bulk of studies do not differentiate between the two isoforms of TSLP, we will also refer to them collectively unless otherwise specified.

## 3. Allergen as a Stimulus for TSLP Release

Alarmin cytokines are overexpressed in the airways of patients with asthma [20], and there are several mechanisms that can drive this response. Exposure of asthmatic airway epithelium to allergenic stimuli induces an increase in alarmin cytokine expression, as evidenced in bronchial biopsies after allergen bronchoprovocation [21,22], and this production of TSLP is associated with infiltration of dendritic cells in allergen-exposed tissue leading to the development of a T2 pro-inflammatory environment [2,23]. 

There are several mechanisms whereby allergenic stimuli induce the production of TSLP. Common aeroallergens, including fungi, dust mites, cockroaches, and pollens, have proteases such as trypsin and papain that activate specific PRR on lung epithelial cells. Proteases found in common allergens have been found to induce TSLP production in human airway epithelial cells via a PRR known as PAR2, a transmembrane G protein-coupled receptor [7,24,25]. Proteases can induce changes in intracellular Ca^2+^ levels by cleaving and activating PAR2 [26,27,28]. Patients with asthma show increased expression of PAR2 on their airway epithelial cells [26], potentially increasing the sensitivity of these cells to proteases in the aeroallergens. In allergic asthma, aeroallergens also activate mast cells through IgE-mediated mechanisms, leading to the release of mast cell-derived proteases such as tryptase, chymase, and carboxypeptidase that can also induce the release of pro-inflammatory cytokines from bronchial epithelial cells [29,30]. 

Changes in the expression transient receptor potential vanilloid 1 (TRPV1) channels may also contribute to increased TSLP production in epithelium in response to allergens. TSLP production relies on calcium influx through the opening pore of the TRPV1 channel and subsequently on the nuclear factor of activated T cells’ (NFAT) translocation from the cytosol into the nucleus [27,31]. Expression of TRPV1 is significantly upregulated in the airway epithelia of patients with refractory asthma [32]. 

In addition to PARs, the ligands for the TLR family, notably, TLR2-6, are expressed by airway epithelial cells. TLRs have been implicated in response to endogenous molecules released by damaged cells or chemical substances generated during tissue injury or inflammation. In fact, crosstalk between PAR2 and TLR4 signal transduction suggests that these receptors may physically interact and cooperate in their inflammatory responses. Concurrent activation of PAR2 and TLR4 by PAR2 and lipopolysaccharide (LPS) amplifies the activation of NFκB [33,34]. Signal transduction of ligands for TLR2, TLR3, TLR8, and TLR9 has all been shown to effectively stimulate TSLP production from airway epithelial cells. Specifically, TSLP induction was shown to be partly dependent on the most well-studied TLR3 ligand when activated by double-stranded RNA [35]. Transcriptional activation of the TSLP promoter is mediated by NFκB after IL-1B and tumour necrosis factor (TNF)-α stimulation [32]. TLR2, TLR8, and TLR9 can induce TSLP expression from airway epithelial cells; the common element in the signaling pathways is the activation of NFκB [36]. TSLP production in the airways from proteases recognized by PAR2 and TLRS associated with airborne allergens facilitate the development and/or exacerbation of T2 airway inflammation in allergic individuals. 

## 4. Viral Infection and TSLP Expression

Viral infections, such as respiratory syncytial virus (RSV) and rhinovirus, are a common cause of asthma exacerbations and are a source of significant morbidity and mortality in asthma [37]. Subjects with asthma have impaired immune response to viral infection, which may be initiated by interactions with a disordered epithelium. In healthy lungs, viruses are taken up by airway epithelial cells via surface proteins and use intracellular machinery for replication [38]. Viral proteins of attachment, cell entry, as well as double-stranded RNA, can be recognized by pattern recognition receptors on endosomes of epithelial cells, such as TLR3 and TLR7, which results in the release of inflammatory cytokines and chemokines: IL-6, IL-8, CCL5, GM-CSF, and interferon (IFN)-γ via transcription factor signaling, notably NFκB, and interferon regulatory factor (IRF) [33,34]. RNA viruses are also recognized by cytoplasmic RNA helicases, such as RIG-1 and melanoma differentiation gene-5, and interferon production is mediated by NFκB [39].

In asthma, there is diminished interferon production and activity in response to respiratory viruses, limiting the primary mechanism of viral incapacitation and leading to increased viral replication [40]. TLR3 expressed on endosomes of airway epithelial cells stimulates TSLP production when activated by double-stranded RNA [10,41]. In bronchial epithelial cultures from patients with allergy, asthma, or chronic obstructive pulmonary disease, exposure to viral RNA or synthetic analogue Poly I:C results in significantly increased production of TSLP, as measured by both mRNA expression and protein [41,42], compared to cells cultured from healthy controls. This elevated TSLP production is directly associated with reduced IFN-β [10,42]. When human bronchial epithelial cells (HBECs) from normal volunteers were simultaneously stimulated with both IL-4 and double-stranded RNA, the production of TSLP was enhanced compared to either stimulus alone. When these stimulated HBECs were placed in co-culture with mast cells, this resulted in enhanced Th2 cytokine production in a TSLP dose-dependent response [43]. This suggests that in the presence of active allergic inflammation, the dysregulated immune response to viral infection becomes more pronounced.

Overproduction of TSLP in asthma epithelium when exposed to viral double-stranded RNA may explain more severe viral pneumonia when compared to patients without asthma, although this response depends on the virulence and features of the virus itself. In contrast to rhinovirus and its synthetic analogues, TSLP is not detected in epithelial cell supernatants or plasma from patients infected with Severe acute respiratory syndrome coronavirus 2 (SARS-CoV-2) [44]. This difference in alarmin activation coincides with the absence of an association between asthma and severe SARS-CoV-2 infection [45]. Furthermore, variation in host-related factors such as pre-existing lung function impairments, active atopy at the time of infection, and even genetic predisposition influences the severity of viral infection and the occurrence of concomitant asthma exacerbation. 

## 5. Air Pollution and Oxidative Stress

**Figure 2:**
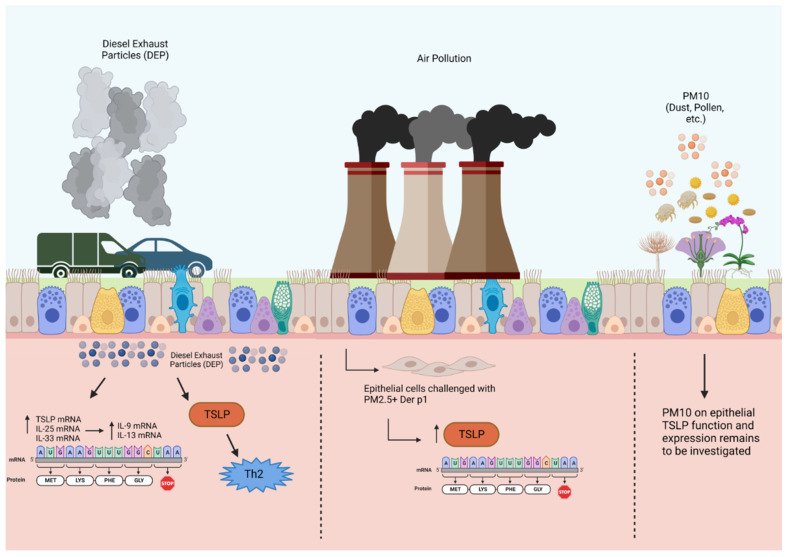
Effects of diesel exhaust particles and particulate matter on TSLP production in airway epithelial cells. Air pollution, including DEPs and PM, causes oxidative stress that upregulates alarmin expression in the airway epithelium through Ahr activation. This upregulation of alarmin mRNA expression in response to DEPs is associated with dendritic cell activity and T2 cytokine expression, specifically IL-9 and IL-13, in bronchial tissues. Particulate matter 10 μm or less in diameter (PM10); particulate matter 2.5 μm or less in diameter (PM2.5). Created with BioRender.com.

Diesel exhaust particles, particulate matter, and environmental pollutants are components of ambient air pollution that have been shown to contribute to allergic asthma and induce asthma exacerbations. DEPs activate Ahr located in the epithelial cell cytoplasm in patients with severe allergic asthma, thereby enhancing mRNA expression of epithelial alarmins in the airways, including TSLP, IL-33, and IL-25 [46]. Chromatin immunoprecipitation assay (ChIP) was used to demonstrate that DEP augments the binding of the Ah/Ahr nuclear translocator complex to alarmin promoter regions. Ahr nuclear translocation in bronchial tissue cells from biopsies was significantly higher in patients with severe asthma [46]. Taken together, these findings suggest that DEP exposure results in epithelial-derived TSLP expression in patients with severe allergic asthma (Figure 2). 

Another pathological component of air pollution is PM2.5, which is characterized by a mixture of harmful inhalable particles less than 2.5 µm in size and generated from the combustion of fuels including gasoline, diesel, wood, and coal [47]. Human bronchial epithelial cells treated with PM2.5 in conjunction with house dust mite antigen significantly increased their production of TSLP and other alarmins, as measured at the protein and mRNA levels, as opposed to HBECs exposed to either stimulant alone [48]. As such, particulate matter exposure in HBECs can drive the innate immune response via alarmins, thus indicating a possible mechanism that may further promote airway inflammation in bronchial asthma. While PM of greater than 2.5 µm (PM2.5–PM10 and PM10) has important clinical implications for asthma patients, its effects on TSLP release have not been studied.

## 6. TSLP Activates Immune Responses

Once released from the epithelium, TSLP binds to its receptors on a broad range of immune cells and activates multiple effector cells involved in the pathogenesis of asthma, most notably dendritic cells (DCs) [49]. Myeloid DCs respond to TSLP by upregulating the co-stimulatory molecules CD40, CD80 CD86, and OX40L, along with IL-8 and eotaxin-2, which lead to the induction of Th2 and Th9 cells and their production of T2 cytokines [14,50,51,52]. TSLP is selective in that it does not stimulate the production of pro-inflammatory cytokines TNF-α, IL-1b, and IL-6, or the Th1-polarizing cytokines IL-12 and type 1 interferons [53]. Recent studies have shown that TSLP also induces CD80 expression in human peripheral blood CD14+ monocytes/macrophages, inducing their activation [54]. During ovalbumin airway challenge in mice, TSLP has been shown to drive the differentiation and activation of alternatively activated macrophages known as M2 macrophages, promoting allergic inflammation in the lung [28]. Moreover, in the presence of TSLP stimulation, natural killer T cells induce the production of the T2 cytokines IL-4 and IL-13 [55]. Additionally, TSLP induces the release of chemokines that attract T cells, including thymus and activation-regulated chemokine (TARC)/CCL17, DC-CK1/pulmonary and activation-regulated chemokine (PARC)/CCL18, macrophage-derived chemokine (MDC)/CCL22, and macrophage inflammatory protein (MIP3β)/CCL19 [56]. 

The alarmin cytokines also drive T2 inflammation through the activation of ILC2 [15,57]. The TSLP/ILC2 axis has been found to play a crucial role in disease, as the addition of TSLP to ILC2s generated from human peripheral blood mononuclear cells (PBMCs) has been shown to mediate resistance to corticosteroids in ILC2s. Furthermore, ILC2s in bronchoalveolar lavage fluid with high levels of TSLP were found to be steroid-resistant, suggesting that the TSLP/ILC2 axis is an important therapeutic target that is not responsive to inhaled corticosteroids, the gold standard in asthma therapy [58]. 

TSLP also affects eosinophils, preventing their apoptosis while upregulating the adhesion molecule CD18, intercellular adhesion molecule-1, and inducing the production of IL-6, CXCL8, CXCL1, and CCL2, collectively promoting eosinophil extravasation and migration to sites of inflammation [59,60]. Mast cells can be stimulated by TSLP to produce cytokines and chemokines, including IL-5, IL-13, IL-6, IL-8, IL-10, GM-CSF, CXCL8, and CCL1, in synergy with IL-1B and TNF-α, while suppressing transforming growth factor (TGF)-β release [11]. On basophils, TSLP can upregulate its own receptor, as well as receptors for IL-25, IL-33, and enhance basophil degranulation [61].

In addition to its effects on the innate immune system, TSLP is also a robust regulator of the adaptive immune system. Despite TSLP not affecting resting CD4+ and CD8+ T cells, following sufficient activation, TSLP signaling directly on naïve T cells in the presence of T cell receptor stimulation promotes the proliferation and differentiation of Th2 cells through the induction of *IL-4* gene transcription [52,62,63]. TSLP can also mediate the proliferation and differentiation of regulatory T cells (Tregs) induced by DCs [64,65]. Furthermore, TSLP directly supports B-cell lymphopoiesis, inducing the proliferation and differentiation of B-cell progenitors [66,67,68]. In the presence of TSLP, multilineage-committed CD34+ progenitor cells, pro B-cells, and pre-B cells differentiate and proliferate [69]. 

## 7. Genetic Variation of TSLP and Relationship to Asthma

GWAS (genome-wide association studies) and gene association studies are two important methods in genetic research used to identify genetic variants associated with certain diseases or traits. Through the examination of many genetic variations across the genome, GWAS can identify genetic variants that may be responsible for complex diseases that are influenced by multiple genes and environmental factors. In contrast, gene association studies focus on a specific gene or a small set of genes that are known to be involved in the disease or trait of interest. Genetic variations within these genes are analyzed to determine whether they are associated with the disease or trait [70,71]. 

Consistent evidence supports that variation in the *TSLP* gene (5q22.1) is related to the risk of asthma in children and adults, as well as other allergic diseases. Two genome-wide association studies conducted in different populations have shown that rs1837253 single nucleotide polymorphisms (SNP) in the *TSLP* gene are associated with increased asthma risk [72,73]. Correlations between SNPs and disease have been described in candidate gene association studies where the CC genotype of SNP rs3806933 was shown to positively correlate with asthma in males [74] (Table 1). 

Genetic variation in TLSP may also be protective. Hunninghake et al. conducted a study showing that two SNPs in the genomic region of *TSLP* (rs1837253 and rs2289276) were inversely correlated with asthma susceptibility [75]. Moorehead et al. also investigated the role of the SNP rs1837253 in *TSLP* gene expression and its association with asthma, allergic disease, and eosinophilia. They reported that individuals carrying rs1837253 was associated with protective effects against asthma, supporting the results of GWAS studies [76]. 

GWAS and gene association studies provide indirect evidence of the role of polymorphisms. However, there are few studies conducted that directly investigate the impact of specific SNPs in controlled environments. In a study of human bronchial epithelial cells examining genetic polymorphism of the *TSLP* gene, long-form TSLP was highly induced by viral double-stranded RNA, and the SNP rs3806933 in the promoter region of long-form TSLP was found to increase promoter–reporter activity of long-form TSLP. Long-form TSLP was shown to enhance binding of the transcription factor AP-1 to the regulatory element, directly in response to viral respiratory infections [16]. The study suggests that the functional variant of the *TSLP* gene contributes to higher TSLP production by bronchial epithelial cells in response to viral stimuli, which could lead to Th2-polarized immunity and increased susceptibility to allergic inflammation [16].

Another study using double-stranded RNA stimulation of epithelial cells was conducted by Hui et al. to examine the effect of the rs1837253 genotype on TSLP secretion from mucosal surfaces. Donors with the minor allele (CT and TT) were shown to have lower TSLP secretion from primary nasal epithelial cells compared to those with the major allele (CC) after stimulation. The study suggests that the rs1837253 polymorphism may be involved in the regulation of TSLP secretion and could help explain the protective association of this genetic variant with asthma and related traits [77]. A similar trend was observed in type 1 inflammation, as noted by Ranjbar et al., where they found that *TSLP* gene SNPs rs2289276 and rs13806933 were associated with decreased serum levels of TSLP in SARS-CoV-2 infected patients [78]. The potential impact of rs1837253 on TSLP production has been a subject of interest. Nakayama et al. investigated the association of the genetic variant rs1837253 with chronic rhinosinusitis with nasal polyps (CRSwNP) and aspirin-exacerbated respiratory disease (AERD) in Japanese populations. The authors found that the susceptible C allele of rs1837253 had a higher binding affinity for transcription factors, including upstream stimulatory factors 1 and 2, which are associated with immune responses following viral and bacterial infections. The study suggests that the rs1837253 variant may contribute to the pathogenesis of CRSwNP and AERD through altered transcription factor binding and subsequent immune response dysregulation [79]. Altogether, the genetic association studies have demonstrated functional differences across *TSLP* gene polymorphisms. However, the question of whether these differences relate to asthma severity or response to asthma medications, such as ICS and anti-TSLP therapy, requires investigation. 

## 8. Clinical Consequences of Targeting TSLP

The prominent pro-inflammatory role of TSLP in the immune response of the epithelium has made it a target of therapy in asthma and other airway diseases characterized by immune dysregulation and epithelial changes. Tezepelumab, a monoclonal antibody targeting TSLP, has been studied in clinical trials of over 1500 patients and has been approved for clinical use in asthma in patients over 12 years of age. 

Proof-of-concept studies have examined mechanisms of TSLP using inhaled allergen challenges in patients with mild allergic asthma. A study evaluating an inhaled TSLP monoclonal antibody fragment (CSJ117) demonstrated that competitive binding of TSLP inhibited allergen-induced bronchoconstriction [80], confirming findings from an earlier study of intravenous tezepelumab [81]. Both anti-TSLP therapies demonstrated attenuation of allergen-induced inflammation after allergen challenge, as measured by sputum eosinophilia and fractional exhaled nitric oxide (FeNO). Tezepelumab also reduced airway hyperresponsiveness and circulating eosinophil levels in mild asthmatic patients. This human monoclonal antibody competitively binds to TSLP and blocks it from interacting with its heterodimeric receptor [82]. 

Clinical trials of subcutaneous tezepelumab, administered monthly in severe asthma patients, demonstrated a significant reduction in the annual rate of exacerbations of up to 60% in the tezepelumab group versus placebo [83]. Prespecified subgroup analysis showed an improvement in the annualized exacerbation rate even in those with low markers of T2 inflammation, such as FeNO less than 25 parts per billion and blood eosinophil count less than 150 cells/µL. Notably, the reduction in the rate of exacerbations was less pronounced than for those with high markers of allergic inflammation at baseline. Therapy with tezepelumab also reduced the rate of exacerbations requiring hospitalization and emergency room visits by 74% in patients without perennial allergen sensitivity. In contrast to other available monoclonal antibodies used in asthma, there was no significant improvement in oral corticosteroid use [84]. With these results and a favorable safety profile, tezepelumab is currently being used for those with severe asthma with a history of exacerbations. Uniquely, it can be prescribed for those who lack allergic inflammation. The effect of extended anti-TSLP therapy on the respiratory epithelium is the subject of ongoing extension trials in severe asthma, which will reveal whether normalizing the alarmin response can improve or prevent the structural changes associated with airway hyperresponsiveness, fixed airway obstruction, and mucous metaplasia. These represent physiologic and histologic targets of asthma remission, but further exploration and study are required.

## 9. Conclusions

The epithelium in allergic airways is a source for TSLP production in response to allergens, viruses, and environmental triggers. In this review, we have identified studies focusing on TSLP in asthmatic airways in an effort to better understand the combined effect of TSLP and epithelial immunology in contributing to disease severity and propagating asthma pathogenesis. The selected GWAS studies have examined how *TSLP* gene mutations associate with asthma susceptibility and regulatory immune responses to allergic stimuli. Studies investigating allergens as stimuli for TSLP release have elucidated the mechanisms of TSLP release in immune cells, emphasizing the importance of PAR2 and TLRs in controlling TSLP production and secretion when orchestrating a T2 response. Heightened epithelial TSLP production during the onset of viral infection is a suggested cause of virus-induced asthma exacerbations in atopic patients; however, this remains yet to be confirmed and requires further investigation. Exposure to low concentrations of particulate matter and DEP has been shown to induce upregulated TSLP expression at the mRNA and protein levels in dendritic cells and HBECs during a T2 response in allergic asthma or in an innate immune response in bronchial asthma. The function of TSLP in response to increased concentrations of air pollutants or synthetic asthma triggers remains to be investigated. With the arrival of anti-TSLP therapy in severe asthma, we will continue to discover the clinical relevance of modulating epithelial responses to inhaled triggers and infectious insults.

## Figures and Tables

**Table 1:** Genetic polymorphisms of TSLP gene associated with asthma.

Genetic Variant	Variant Type	Correlation with Asthma Susceptibility
rs3806933	SNP	(+) Higher risk in males
rs1837253	SNP	(−) Lower risk
rs2289276	SNP	(−) Lower risk
